# El sistema de salud en Colombia 1945-2020: entre la realidad y el imaginario social

**DOI:** 10.15446/rsap.V25n2.106502

**Published:** 2023-03-01

**Authors:** Luis Gonzalo Morales-Sánchez

**Affiliations:** 1 LM: MD. M. Sc. Salud Pública. M. Sc. Ciencia Política. Esp. Administración. Esp. Economía. Medellín, Colombia. luisgmoral@yahoo.com Esp. Administración Esp. Administración Medellín Colombia

**Keywords:** Políticas de salud, seguridad social, políticas públicas, capitalismo, socialismo, política *(fuente: DeCS, BIREME)*, Health policy, social security, public policy, capitalism, socialism, politics *(source: MeSH, NLM)*

## Abstract

Este ensayo describe los principales hechos sociales, políticos y económicos del mundo entre 1919 y 1993 que enmarcaron el desarrollo de la protección social en salud en Colombia. Al Tratado de Versalles al final de la Primera Guerra Mundial en 1919, que creó la Organización Internacional del Trabajo (OIT), le siguieron la Gran Depresión mundial en 1929 y la Segunda Guerra Mundial en 1939. Luego, se inició (1945) un período de crecimiento económico conocido como los Estados de bienestar, que finaliza con la crisis mundial del petróleo en 1973, ligada al surgimiento del neoliberalismo. China y la Unión de Repúblicas Socialistas Soviéticas (URSS) también se analizan como formas de organización social no capitalista. En Colombia, la protección social en salud comenzó con la creación del Ministerio de Salud y los Seguros Sociales (1945), continuó con el Sistema Nacional de Salud (1976) y finalizó con el Sistema General de Seguridad Social en Salud (1993), que algunos califican de receta neoliberal y retroceso social, lo que sigue siendo motivo de debate.

## Abstract (en)

This essay describes the main social, political, and economic world events between 1919 and 1993 that encompassed the development of social health protection in Colombia. In 1919, the Treaty of Versailles, conceived at the end of World War I, created the International Labor Organization. After the Great Depression in 1929 and the World War II in 1939, followed a period of economic growth known as the Welfare State (circa 1945), ending with the world oil crisis in 1973, from which it is believed to have emerged Neoliberalism. China and the Union of Soviet Socialist Republics (USSR) are also studied as non-capitalist social organization systems. In Colombia, social health protection began in 1945 with the creation of the Ministry of Health and Social Security, followed by the National Health System in 1976, and more recently, the General Social Security System for Health in 1993, which some call a neoliberalist formula that unchained social regression, which remains up for debate.

Este ensayo analiza los principales hechos de carácter social, político y económico ocurridos en el mundo entre 1919 y 1993 que enmarcaron el desarrollo de la protección social en salud en Colombia. Tales hechos comenzaron en 1919 con el Tratado de Versalles, al final de la Primera Guerra Mundial, que creó la Organización Internacional del Trabajo (OIT), la cual resaltó la justicia social como condición para una paz universal y duradera 1. Continúa con la Gran Depresión económica mundial de 1929, iniciada en Estados Unidos, que junto con la Segunda Guerra Mundial motivó el desarrollo de los llamados Estados de bienestar en el mundo capitalista occidental a partir de 1945 2,3. En Colombia, coincidió con la creación de los ministerios de Salud y los Seguros Sociales desde 1945, lo que marcó el inicio de la protección social en salud 4,5. Este fue un período de treinta años de crecimiento económico jalonado por la reconstrucción de la Europa de la posguerra, que finalizó con la recesión económica mundial desatada por la crisis del petróleo en 1973 6,7. China y la Unión de Repúblicas Socialistas Soviéticas (URSS) también se analizan como sistemas de organización social no capitalista 8,9. La crisis del petróleo se relaciona con la aparición del neoliberalismo como ideología política 10,11, que algunos creen que influyó en el desarrollo del sistema de salud en Colombia, en especial a partir de la reforma de 1993 12-16.

Con el análisis de los principales sucesos mundiales, en el marco de los cuales se desarrolló el sistema de protección social en salud en Colombia, se espera tener argumentos para poder ir más allá de las recientes discusiones ideológicas que han caracterizado el abordaje de este tema.

## La Primera Guerra Mundial

La Primera Guerra Mundial, que involucró a las grandes potencias mundiales del momento, finalizó con la firma del Tratado de Versalles en 1919. Este hecho dio lugar a la creación de la Sociedad de las Naciones y a la Comisión Internacional del Trabajo, que destacó a la justicia social como elemento central para lograr una paz universal y duradera 1. En 1927, esta comisión sugirió el establecimiento de un seguro contra la enfermedad que cubriera a los obreros, los empleados y los aprendices; al tratamiento médico y el suministro de drogas gratuito; a contar con entidades públicas especiales autónomas y sin fines de lucro para la atención a la enfermedad financiadas por el Estado, los trabajadores y los patronos 17. Este tratado fue ratificado por Colombia en 1930, durante la presidencia del liberal Enrique Olaya Herrera; no obstante, su inicio fue postergado hasta después de 1940 por la recesión económica de 1929 y la Segunda Guerra Mundial en 1939 5.

## La gran depresión de 1929

La Gran Depresión económica de 1929 se inició en los Estados Unidos y luego se propagó por todo el mundo 18. Esta fue una crisis social y económica profunda, severa y prolongada, originada por diversas causas. Para el Nobel de Economía Milton Friedman, la principal causa estuvo en la equivocada política monetaria de la Reserva Federal de los Estados Unidos, que redujo en un tercio la cantidad de dinero circulante, lo que generó escasez de recursos y convirtió una crisis local en una recesión mundial 19. Esta depresión se caracterizó por una caída del 38 % en los precios; el cierre masivo de empresas, entre las cuales las más afectadas fueron las de la construcción, las de la agricultura y las de la industria del automóvil; la quiebra del sistema bancario; un desempleo que pasó del 2,9 % en 1929 al 22,9 % en 1932; y una caída del 35 % en el ingreso per cápita. Esta situación terminó afectando a Europa, con diferentes grados de intensidad y duración, por efecto de la menor demanda de bienes y servicios importados por Estados Unidos. Muestra de ello es que mientras en 1929 el valor de las exportaciones de Estados Unidos ascendió a 7 billones de dólares, en 1932 cayeron a 2,5 millones; y las importaciones pasaron en el mismo período de 5,9 a 2 billones de dólares 20.

## Los estados de bienestar en 1945

Los Estados de bienestar se asocian al período de treinta años de crecimiento económico en el mundo capitalista en América y Europa, cuyo pilar fundamental fue la protección social 3. Este período comenzó en 1945 con el final de la Segunda Guerra Mundial y culminó a mediados de 1970 con la recesión económica global desatada por el incremento de los precios del petróleo 6,18. Los Estados de bienestar tuvieron al capitalismo como modo de producción, basado en la libertad individual, la democracia como forma de organización social, y el mercado, combinado con la acción estatal, como herramientas para garantizar la estabilidad económica y el progreso social 7.

Previamente, se habían desarrollado experiencias de protección social, como la de Alemania en el siglo XIX, con un programa de protección a los trabajadores por iniciativa del canciller Otto von Bismarck 7. En Inglaterra, durante la Segunda Guerra Mundial, con el liderazgo de William Beveridge, se diseñó un sistema de protección social que cubría a toda la población, con independencia de su capacidad para pagar los servicios 21.

Esta época fue un marco de discusiones económicas, políticas y sociales acerca del papel de la acción estatal y el mercado en la búsqueda del bienestar, que cobraron relevancia en 1930 con las doctrinas económicas de Jhon Maynard Keynes, considerado el teórico del Estado de bienestar en Estados Unidos. Sus ideas enfatizaban en la función del Estado en el manejo de la economía, que debería encargarse de promover el desarrollo, controlar el desempleo y redistribuir el ingreso por la vía de las políticas sociales 3. Esta época coincidió con el ascenso al poder en Estados Unidos del demócrata Franklin Delano Roosevelt en 1933 22, cuyo partido históricamente ha sido considerado de ideas más progresistas en ese país.

## La crisis de los estados de bienestar 1973

El aumento de los precios del petróleo en 1973, ordenado por la Organización de Países Exportadores de Petróleo (OPEP), desató una nueva recesión económica mundial 6 . Esta facilitó el ascenso al poder gobiernos conservadores, mucho más proclives a las ideas económicas de Milton Friedman, que no obstante poner un especial énfasis en la política económica, no negaban ni proscribían la necesidad de la intervención del Estado. Para esa época fue elegida la conservadora Margaret Thatcher en Inglaterra en 1979, y el republicano Ronald Reagan en Estados Unidos en 1981, conocidos como los gobernantes más emblemáticos de las políticas económicas monetaristas 7. A este tiempo y a estos gobernantes se les relaciona con el origen del neoliberalismo como ideología política, construida por sus detractores, que a su juicio apelaba al libre mercado extremo sin la intervención del Estado 10,11, que algunos consideran influyó en el desarrollo de los sistemas de protección social, en especial en la reforma de Colombia en 1993 12-15.

## La idea del bienestar en china y la urss

Con el triunfo de la revolución bolchevique en 1917, encabezada por Vladimir Ilich Lenin, se conformó la Unión de Repúblicas Socialistas Soviéticas (URSS). De otro lado, la derrota de Chiang Kaishek en China en 1949 produjo el ascenso al poder del comunismo, en cabeza de Mao Zedong 8. Ambas naciones surgieron como alternativas de producción y conducción de la sociedad, en contraposición a la iniciativa individual, la propiedad privada y la democracia que caracterizaban a los Estados capitalistas de Occidente. Estos aspectos serían sustituidos por el Estado centralizador y autoritario en su función de regular las relaciones sociales y económicas 7. Aunque en estos países no se formaron Estados de bienestar como los de Occidente, la promesa de bienestar fue similar o quizás superior. Esto originó la confrontación ideológica entre capitalismo y socialismo como formas opuestas para alcanzar el bienestar, fundamentadas en teorías sociales, económicas y políticas disimiles 8.

Para la década de 1970 estos países también experimentaron severas crisis, en parte por las razones de Occidente y en parte por la creciente demanda de mayores libertades, apertura y transparencia de sus gobiernos. Lo anterior, motivado además porque no había ocurrido el prometido bienestar, el crecimiento de la riqueza ni su redistribución. En China, por ejemplo, dos tercios de la población continuaban viviendo en condiciones de pobreza. Estos hechos se constituyeron en una crítica de fondo al socialismo que surgió en su interior mismo, como modo de producción y gobierno de la sociedad, que llevó a dichos Estados a buscar otras alternativas para salir del atraso social y económico 7. El final se dio en la Unión de Repúblicas Socialistas Soviéticas (URSS), encabezada por Mijail Gorbachov, con la aparición de las políticas del glásnost, que significaba transparencia, y la perestroika, como la apertura económica y social hacia Occidente, que concluyeron en la disolución del Estado en 1991 y la conformación de quince Estados independientes, la mayoría de los cuales adoptaron el capitalismo y la democracia. Por otro lado, el Estado comunista chino terminó con la muerte de Mao Zedong en 1976 y el ascenso al poder en 1978 de Deng Xiaoping, conocido como el arquitecto del Estado chino moderno, pero que, a diferencia de la Unión Soviética, continuó siendo una sola nación controlada por el Partido Comunista 8.

El fin de los Estados socialistas podría haber significado la hegemonía total del capitalismo 7, al desaparecer su contraparte ideológica por el fracaso del modo de producción socialista y la adopción del capitalismo en su reemplazo. No obstante, al menos en China, esto no ocurrió en su forma de organización social, que siguió siendo de control centralizado y autocrático. Esto explica lo que ha venido sucediendo en China, hoy primera potencia económica mundial, basada en una apertura a la iniciativa privada y al intercambio comercial con Occidente como motores de su desarrollo, algo impensable antes de 1976 9.

## Los estados de bienestar en latinoamérica

Aunque existe literatura sobre diversos programas sociales desarrollados en la región a partir de 1945, esta se ha enfocado más en analizar situaciones específicas de manera aislada y no como Estados de bienestar o sistemas de protección social. Se destaca el concepto de Estado desarrollista de la Comisión Económica para Latinoamérica y el Caribe (Cepal), bajo la conducción de Raúl Prebisch, basado en la industrialización por sustitución de importaciones, del que las políticas sociales hacían parte, pero sin estar explícitamente definidas, modelo que también llegó a su fin con la crisis del petróleo de 1973. A partir de ese momento se inició en la región una prolongada y severa crisis social y económica, agravada por la subsiguiente crisis de la deuda externa en 1982 que, como ya se señaló, algunos autores asocian al surgimiento de las recetas neoliberales para conjurarla 12-15 y que según ellos fueron determinantes en el desarrollo de la protección social en salud en Colombia, como se describirá enseguida 2,6,23,24.

## La protección social en salud en colombia

En Colombia, la protección social comenzó a consolidarse a partir de 1945; antes solo habían existido puntuales decisiones estatales o del sector privado. Se destaca como la primera el establecimiento de los montepíos militares en 1761, mediante real cédula del rey Carlos III, instituciones del Estado español cuya función era proteger a las viudas y a los huérfanos de los militares, y que operaron hasta 1860. Luego, la Constitución de 1886 estableció que la salubridad pública sería una responsabilidad del Estado, y ese mismo año se expidió la Ley 30, que creó las Juntas de Higiene Nacional y Departamental, antecesores del Ministerio de Salud 4,5.

En 1904 se le atribuyó al general Rafael Uribe la idea de un código laboral, de seguros sociales y de las cajas de ahorros y vivienda de los trabajadores. Entre aquel año y 1941 se presentaron al Congreso una serie de iniciativas legislativas para crear elementos de la protección social, algunas de las cuales eran iniciativas puntuales o aisladas, y fueron rechazadas, no se pusieron práctica, o nunca se formalizaron como políticas generales 4,5.

En 1945 y 1946 se dio comienzo a la estructuración formal de los modelos de protección social en salud con un alto grado de sistematización 4,5, dentro de los cuales se pueden identificar las siguientes tres etapas en su desarrollo:

- Los Seguros Sociales para los trabajadores formales. Se inició en 1945 con la Ley 6.a, que creó la Caja Nacional de Previsión Social (Cajanal), y en 1946, con la Ley 90, que creó el Instituto Colombiano de los Seguros Sociales (ICSS), para cubrir a los trabajadores del sector público y del sector privado, respectivamente. Este consistió en la creación de entidades especiales en las que participarían en su administración y financiación el Estado, los empleadores y los trabajadores, que se encargarían de cubrir los riesgos de enfermedad, discapacidad, vejez y muerte derivados de circunstancias comunes o de las asociadas al trabajo 5, tal como lo había establecido la Comisión Internacional del Trabajo en 1927. El resto de la población, los trabajadores del sector informal y los pobres seguirían siendo atendidos por la asistencia pública, en cabeza del Ministerio de Salud, con los hospitales públicos a su cargo, en tanto que las personas con capacidad acudirían al sector privado. El Seguro Social, desde su creación y hasta 1975, tuvo una época de expansión de la afiliación y de construcción de instalaciones de salud, pero desde 1970 se estancó en su cobertura, que nunca logró superar el 25 % de la población 25.
- El Sistema Nacional de Salud. Se inició con la expedición del Decreto-Ley 654 de 1974, que tuvo continuidad con el Decreto 56 de 1975, el cual creó el Sistema Nacional de Salud, conformado por tres subsistemas. El principal objetivo de este nuevo esquema fue lograr integrar en una sola estructura y bajo la dirección única del Ministerio de Salud los tres subsectores de la protección social: el Seguro Social, la asistencia pública oficial con los hospitales públicos y el creciente sector privado de hospitales y aseguradoras de salud, la primera de las cuales se creó en 1980. Esta idea no pudo ponerse en marcha dada la irreconciliable rivalidad de intereses políticos, económicos y sindicales que impidieron que el Ministerio de Salud asumiera la conducción del sistema. Posteriormente, se dieron los primeros pasos para desconcentrar y descentralizar en municipios y departamentos el manejo de la salud y los hospitales públicos, bajo el esquema promovido por la Organización Panamericana de la Salud, conocido como Sistemas Locales de Salud (Silos), mediante la aprobación de la Ley 10 de 1990, proceso que se fortaleció con la expedición de una nueva Constitución en 1991 25.
- El Sistema General de Seguridad Social en Salud. Esquema vigente en Colombia en la actualidad, comenzó con la aprobación de la nueva Constitución Política en 1991, cuyo artículo 48 dispuso que la seguridad social sería un "derecho inalienable de todos los colombianos" y "una responsabilidad del Estado", lo que desvirtúa por completo el supuesto influjo neoliberal; esto se materializó con la expedición de la Ley 100 en 1993 5. Igualmente, el artículo 86 de la nueva Constitución creó la tutela como mecanismo de amparo constitucional expedito de ciertos derechos, entre los cuales más tarde se reconoció el derecho la salud. A partir de ese momento, desaparecieron los tres subsectores que habían surgido en 1945, y comenzó el desarrollo de un esquema único que debería cubrir a toda la población, en el que la afiliación y el acceso a un amplio paquete de beneficios, ya no serían diferenciales, ni estarían sujetos a la capacidad de pago de las personas o a su condición laboral, y cuya financiación debería ser una obligación del Estado, modelo que adoptaba varias de las premisas del modelo Beveridge inglés (21,24). Este ha tenido varios ajustes, entre los que se destacan las Leyes 1122 del 2007, 1438 del 2011, 1751 de 2015 o ley estatutaria de salud, y 1753, que creó la Administradora de Recursos del Sistema (Adres). A día de hoy, la cobertura financiera del riesgo de enfermar alcanza un 100 %, y en cuanto al acceso a los servicios, aunque ha mejorado sustancialmente, en especial en las zonas urbanas, persisten dificultades en las zonas rurales 26,27.

## Reflexiones finales

Los principales hechos de carácter social, político y económico ocurridos en el mundo entre 1919 y 1993 han conducido, de una u otra forma, a la necesidad de la justicia y el bienestar social, que son los pilares fundamentales para lograr una paz estable y duradera, única forma de alcanzar el progreso en cualquier sociedad. No obstante, este no ha sido un proceso exento de controversias, en especial cuando se trata de encontrar la mejor forma de alcanzar estos bienes sociales supremos. A partir de este momento surgen las contradicciones acerca de las formas de producción y organización social que se deberían utilizar para alcanzarlos, que originan la confrontación ideológica entre capitalismo o socialismo, Estado o mercado, política social o política económica. Ante esta aparente dicotomía, valdría la pena reflexionar si es posible una sociedad en la que solo existan el Estado o el mercado como formas únicas para alcanzar el bienestar social. La evidencia sugeriría que no, que ambos son inseparables, necesarios e interdependientes. La discusión radicaría más bien en el grado en el que uno y otro se conjugan dependiendo de los contextos, sin que sea posible negar la existencia de ninguno de los dos o definir reglas fijas de la mezcla ideal que deberían tener. Por ello, resulta también discutible darles calificativos éticos que lleven a pensar que el uno es bueno y el otro malo, o que se pueda subordinar o subsumir la importancia del uno con relación al otro. De otro lado, el avance alcanzado por la protección social en salud en Colombia, en especial después de la Constitución de 1991 y la Ley 100 de 1993, dista mucho de poder ser considerado una política neoliberal que le hubiese dado mayor peso al mercado, y menos aún que haya generado un retroceso social. Finalmente, faltaría por discutir cuál debería ser la mejor forma de organización social para acompañar la mejor forma de producción social. Aunque parecieran no tener relación, la evidencia también ha demostrado que son igualmente aspectos inseparables e interdependientes, discusión que no es objeto de este ensayo ♦
